# Looking into the Black Box: Insights into the Mechanisms of Somatic Cell Reprogramming

**DOI:** 10.3390/genes2010081

**Published:** 2011-01-13

**Authors:** Laurent David, Payman Samavarchi-Tehrani, Azadeh Golipour, Jeffrey L. Wrana

**Affiliations:** 1 Center for Systems Biology, Samuel Lunenfeld Research Institute, Mount Sinai Hospital, 600 University Ave. B 119, Toronto, ON M5G 1X5, Canada; E-Mails: ldavid@lunenfeld.ca (L.D.); payman@lunenfeld.ca (P.S.-T.); golipour@lunenfeld.ca (A.G.); 2 Department of Molecular Genetics, University of Toronto, Canada

**Keywords:** induced pluripotent stem cells, mechanism of reprogramming, iPS, BMP, Wnt, p53, epigenetic, MET, PKA, histone modifications

## Abstract

The dramatic discovery that somatic cells could be reprogrammed to induced pluripotent stem cells (iPSCs), by the expression of just four factors, has opened new opportunities for regenerative medicine and novel ways of modeling human diseases. Extensive research over the short time since the first iPSCs were generated has yielded the ability to reprogram various cell types using a diverse range of methods. However the duration, efficiency, and safety of induced reprogramming have remained a persistent limitation to achieving a robust experimental and therapeutic system. The field has worked to resolve these issues through technological advances using non-integrative approaches, factor replacement or complementation with microRNA, shRNA and drugs. Despite these advances, the molecular mechanisms underlying the reprogramming process remain poorly understood. Recently, through the use of inducible secondary reprogramming systems, researchers have now accessed more rigorous mechanistic experiments to decipher this complex process. In this review we will discuss some of the major recent findings in reprogramming, pertaining to proliferation and cellular senescence, epigenetic and chromatin remodeling, and other complex cellular processes such as morphological changes and mesenchymal-to-epithelial transition. We will focus on the implications of this work in the construction of a mechanistic understanding of reprogramming and discuss unexplored areas in this rapidly expanding field.

## 1. Introduction

Pluripotent Embryonic Stem cells (ESCs), isolated from the inner cell mass (ICM) of a blastocyst, have the potential to differentiate to the three germ layers. Conversely, a differentiated somatic cell can be reverted, or reprogrammed, to a pluripotent state. Somatic cell reprogramming has been accomplished through three major methods. Classical methods have involved: (1) transfer of the nucleus of a differentiated cell to an enucleated oocyte; and (2) cell fusion of differentiated cells with ESCs [1]. The success of these approaches stimulated the hunt for molecular factors present in stem cells that might drive reprogramming. This led to the discovery of the third approach, where expression of just four embryonic stem cell factors, Oct4, Klf4, c-Myc and Sox2 (OKMS), was sufficient to induce fibroblasts to assume an embryonic stem cell phenotype [2]. Such pluripotent stem cells are called induced pluripotent stem cells (iPSCs) and were shown to contribute to all cell lineages in the mouse, including the germ cells. In terms of both technical difficulties and ethical concerns, factor-based reprogramming is more advantageous than the two other reprogramming methods.

Aside from the mouse ESC derived from the ICM, there are a number of other pluripotent cell types that have been identified and are thought to reside in metastable states; that is, different stable pluripotent states that can be interconverted [3]. For example, cells derived from the mouse epiblast at E5.5, termed Epi Stem Cells (EpiSCs) [4,5], display a lower pluripotent potential in comparison to mouse ESCs. Interestingly, human ESCs are functionally closely related to mouse EpiSCs, with both stem cell types employing TGFβ and FGF2 signaling to maintain their pluripotent state. More recently, treatment of human ESCs or iPSCs by Leukemia Inhibiting Factor (LIF) along with drugs (Forskollin and inhibitors of GSK3β and MEK), or over-expression of pluripotency-related transcription factors (OKMS plus Nanog), has yielded human cells that have mouse ESC like morphology and have been dubbed mouse ES-like human ESCs [6–8].

**Figure 1:**
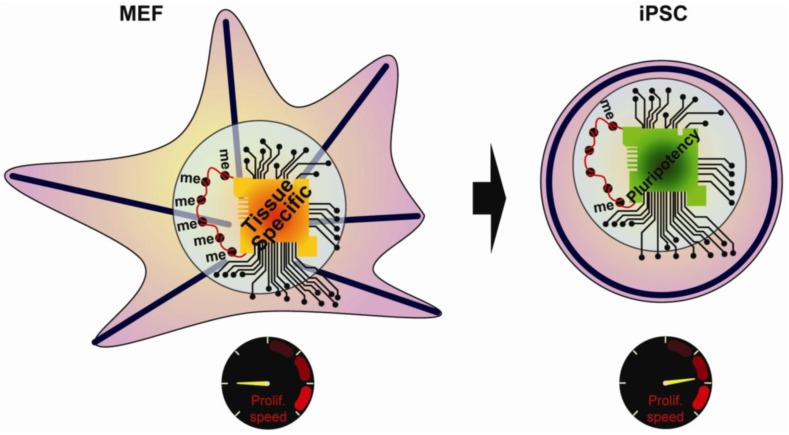
During reprogramming, the MEF have to increase their speed of proliferation, remodel their actin network (blue line), change their transcriptionnal circuitry and reactivate their chromatin.

The early studies on reprogramming resulted in a number of key observations, including changes in the cellular morphology, the chromatin state, the transcriptional regulatory network, and the proliferation rate (Figure 1). Despite clear characterization of the starting cell type and the final iPSCs, the temporal sequence of events and the mechanisms underlying reprogramming have remained ambiguous. However, secondary reprogramming systems that express reprogramming factors from inducible promoters have recently emerged and display much higher efficiency compared to primary systems. In secondary systems, primary iPSCs are first generated using inducible reprogramming factor transgenes. These primary iPSCs are then differentiated either *in vivo* via the generation of chimeric mice, or *in vitro*, via differentiation of primary iPSCs [9–12]. An alternative approach uses reprogrammable mouse lines obtained after integration of inducible factors into the genome of ESCs [12,13]. Subsequent re-induction of the transgenes gives much higher reprogramming efficiency compared to primary systems, thus allowing for a detailed study of the sequence of regulatory events underlying reprogramming. The focus of this review will be on recent work aimed at defining the mechanisms that take place during reprogramming, and highlight those that are similarly important for stem cell maintenance or primordial germ cells (PGCs) specification.

## 2. Processes Regulating Induced Pluripotency

### 2.1. Regulation of Induced Pluripotency by Cell Growth

The growth of a cell population depends on the balance between cells that are actively cycling and dividing, and cells that are senescing or dying. Here we will discuss how proliferation and senescence/apoptosis affect reprogramming. The first visible characteristic gained by somatic cells on the road to pluripotency is an increase in proliferation rate; all major reprogramming cocktails contain proliferative factors such as c-Myc or Lin28 alongside Oct4, Sox2, Klf4 or Nanog [2,14]. c-Myc is a transcription factor that directly induces the expression of multiple genes involved in cell cycle progression. Lin28, a pluripotency associated transcription factor used for reprogramming human cells, stimulates proliferation through inhibition of Let-7 miRNA, which in turn represses c-Myc [15]. Analysis of gene expression profiles of reprogrammed cells compared to parental cells has further revealed the strong up regulation of the Cyclins *Ccnd1* and *Ccnd2*, which coordinate cell cycle progression [16]. Moreover, using secondary pro-B cells as a model system, Hanna and colleagues showed that induced pluripotency depends on the number of cell divisions [17]. They hypothesized that a low-frequency epigenetic event is required for reprogramming, therefore the number of cell cycles increases the odds of this event occurring.

Aging of cells or genotoxic stress activate signaling cascades that will block cell cycle progression, leading to cellular senescence or apoptosis. Cells that have escaped senescence, by spontaneous immortalization or expression of SV40 large T antigen, reprogram more efficiently, suggesting that senescence hinders reprogramming [18,19]. Indeed, MEFs nearing senescence (late passage) are poorly reprogrammed, and the microRNA families miR-290 and miR-302, which inhibit senescence by inhibiting the expression of G1/S checkpoints, improve their reprogramming [20,21]. Chromatin immunoprecipitation (ChIP) analysis further revealed that these miRNAs are directly induced by c-Myc and can replace c-Myc during reprogramming of MEFs [20]. This indicates that c-Myc may fulfill a dual role during reprogramming: Promoting proliferation while suppressing senescence.

Reprogramming factors cause the induction of certain apoptosis signals through accumulation of DNA damage and oxidative stress [22,23]. One of the main events triggered by DNA damage is p53 expression [24], which leads to senescence, and to apoptosis through the Bax/Bcl2 pathway. Multiple groups demonstrated that inhibiting the senescence/apoptotic response through knock down of p53, or components of the p53 pathway, improves reprogramming, reinforcing the central role for p53 in controlling senescence throughout reprogramming [22,23,25–28]. Furthermore, knock down of p21, a main downstream target of the p53 pathway, resulted in an increase in reprogramming efficiency [26,27]. Among the upstream components of p53 signaling pathway, the *lnk4/Arf* locus was described to regulate reprogramming [19,22,27]. This locus encodes three tumor suppressors: p19 (Arf), p16 and p15 (Ink4a and b). p16 and p15 inhibit Cdk4 and Cdk6, which are critical to relieve the anti-proliferative pressure of Rb; p19 inhibits Mdm2, the main ubiquitin ligase targeting p53 for degradation [29]. It is interesting to note that the *Ink4/Arf* locus is silenced in mouse iPSCs and ESCs. Consistently, knock-down of the *Ink4/Arf* locus in MEFs accelerated the reprogramming process and increased the number of successfully reprogrammed cells [19,22,27].

Mechanistically, the induction of senescence by OKMS was hypothesized to be the result of DNA-replication-induced DNA damage [28]. c-Myc, however, was shown, in human fibroblasts, to predominantly induce DNA damage through accumulation of radical oxygen species (ROS) [30]. Accordingly, decrease of oxidative stress through low-oxygen culture conditions [19,31] or vitamin C treatment [32] improved the reprogramming efficiency of MEFs through anti-senescence effect, by limiting the accumulation of ROS, therefore preventing the up-regulation of p53 [24]. Interestingly, anti-oxidant effects were shown to be important to preserve both activated X chromosomes during the derivation of human ESCs [33], suggesting other mechanisms might link the oxidation state of the cells and their pluripotency potential.

Collectively, these data suggest that in order for reprogramming to occur, it is essential to overcome senescence and apoptosis induced by the reprogramming factors themselves. In this context, it is important to remember that either loss of p53, or gain of c-Myc, are events that promote cell transformation. In fact, p53 knocked-down iPSCs were more likely to give rise to chimerae that developed tumors and died after seven weeks [26]. Consistent with this, a high tumor rate associated with a loss of senescence control was previously observed in chimerae arising from c-Myc retrovirus-containing iPSCs [34]. Thus, a key goal, for development of safe applications of iPSC technology in the clinic, is to balance proliferative requirements for achieving iPSCs, without increasing the risks of tumor formation.

### 2.2. Resetting the Epigenome during Reprogramming

Epigenetics encompasses a broad range of chromatin modifications, such as DNA methylation, covalent histone modifications, nucleosome organization and higher order chromatin structures, all of which impact transcriptional events to affect cell fate. Although the genome of somatic cells is mostly in a heterochromatic (transcriptionally repressive) conformation, the genome of stem cells is predominantly in a euchromatic (transcriptionally permissive) conformation [35]; thus there are a number of epigenetic modifications associated with reprogramming such as DNA methylation, histone modifications, chromatin remodeling, X-reactivation, silencing of the retroviral promoters, and genomic imprinting. Given the diversity in epigenome modification and regulation; targeting this complex process using small molecule compounds has been a major focus for enhancing reprogramming efficiency [36,37].

Transfer of mouse *nuclei* in *Xenopus* Oocytes has highlighted the importance of resetting the epigenome to that of ESCs for successful nuclear reprogramming [38]. Expression profiles, histone marks and methylation patterns of iPSCs compared to ESCs reveals strong similarities [16,39–43], but despite this, expression profiling has nonetheless revealed that the cell of origin used for reprogramming contributes to the transcript repertoire of human iPSCs [44]. By generating iPSCs from cells belonging to different lineage, using an inducible secondary reprogramming system, two groups demonstrated that iPSCs retain some transcriptional memory of the somatic cell of origin [45,46]. CpG methylation differences in the iPSCs could distinguish between the tissues of origin of the iPSCs, ESCs, or nuclear transfer derived ESCs (ntESCs). Moreover, the epigenetic memory coincided with differentiation outcomes, with ESCs and nuclear transfer derived ESCs having the broadest potential, while iPSCs generated from fibroblasts or the blood lineage have a bias in differentiation potential toward their cell lineage of origin [45,46]. This is consistent with nuclear transfer experiment done in Xenopus: Ng and Gurdon observed that when embryos were obtained from nuclear transfer of neuroectoderm nuclei that express high levels of Sox2, subsequent analysis of Sox2 revealed ectopic expression in all tissue, not just neuroectoderm. They further concluded that has to be due to epigenetic memory [47]. Thus, the retention of the original epigenetic marks on a small subset of genes during reprogramming likely leads to subsequent aberrant expression patterns reprogrammed stem cell offspring.

More recently, ways to reset epigenetic memory have been discovered. Polo *et al.* found that methylation differences at lineage specific loci subtly contrasted more dramatic differences observed at the transcript level [45]. However, further passaging of these iPSCs led to normalization of these epigenome differences and equalized the differentiation potential of the different iPSC clones [41,45]. Use of tertiary reprogramming, lineage specific differentiation, or use of drugs that destabilize the epigenome (TSA or AZA), can also erase iPSCs' epigenetic memory [46]. Here, we will discuss the mechanisms involved in resetting various aspects of epigenetic memories.

#### 2.2.1. DNA Methylation

DNA methylation predominantly occurs at cytosine of CpG dinucleotides. The distribution of the CpG dinucleotides falls into regions of high, medium and low density; the former two are termed, respectively, CpG islands and island-shores. These modifications are catalyzed by DNA methyltransferases: Dnmt1, which maintains methylation, and Dnmt3a/b, which induces *de novo* methylation. On the other hand, erasure of DNA methylation occurs at a number of pluripotency related genes during reprogramming and is mediated by demethylases or by replacing the methylated with un-methylated cytosines via deamidase activity [48,49]. Indeed, small molecule DNA methyltransferase inhibitors such as RG108 [50] and AZA [51], accelerate reprogramming. Additionally, AZA can be used late in reprogramming to rescue partially reprogrammed cells (pre-iPSCs), a result that was recapitulated through the knockdown of Dnmt1 [16]. Although demethylases have not yet been identified in mammals, the role of deamidases, which function upstream of the Base Excision Repair (BER) pathway, has been examined during reprogramming. Using interspecies heterokaryons (mouse ESCs fused with human fibroblasts), which reprogram without cell division and in one day, Bhutani *et al.* showed that AID, a 5-methyl-cytosine deaminase, was required for promoter demethylation and induction of Oct4 and Nanog expression [52]. AID binds to methylated Oct4 and Nanog promoters in human fibroblasts and is part of a complex of enzymes in charge of demethylation that has yet to be fully resolved. The role of the BER pathway was further highlighted in the context of PGCs specification. The PGCs need a profound remodeling of their genome to enable production of gametes. Hajkova *et al.* showed that during mouse development the BER was essential to achieve proper genomic rearrangement [53]. This supports the hypothesis that demethylation in mammals is achieved through deamination of cytosine, creating a T-G mismatch that will then be repaired by the BER complex [54].

#### 2.2.2. Histone Modifications

The fundamental structure of the chromosome consists of DNA wrapped around the core histone octamer. The *N*-terminal tails of histones, protruding out of the structure, are subject to various forms of genome-activity modulating modifications, including acetylation, regulated by Histone Acetyl Transferase (HAT) and Histone Deacetylases (HDAC), and methylation, regulated by Histone Methyl Transferases (HMT) and Histone Demethylase (HDMase). The combinatorial function of the covalent histone modifications constitutes the “histone-code”. Histone acetylation (H3K4Ac) is generally a mark of active transcription, while the effect of methylation depends on the residue on which it is placed; H3K4me3 are marks of active transcription, while H3K27me3 and H3K9me2/3 are repressive marks, with the latter of the two being a stable repression and heterochromatin mark [55]. Traditionally, it was thought that active and repressive histone marks were distributed across the genome in a mutually exclusive manner. However, analysis of genome-wide histone modifications led to the discovery of regions of chromatin that harbor both marks, which are termed “bivalent domains” [56]. In fact, in ESCs and iPSCs, three-quarter of the H3K27me3 repressive domains contain H3K4me3 active domains within them. These bivalent domains generally coincide with developmentally important genes that are expressed at low levels in ESCs. Upon differentiation, the bivalent domains resolve to either the monovalent state of repressive H3K27Me3 or active H3K4Me3 [16,56].

In their original description of factor-based reprogramming, Takahashi and Yamanaka observed an increase in H3K4Ac and a decrease in the repressive mark H3K9me2 at the Oct4 and Nanog promoters, although these promoters had not lost their CpG methylation [2]. They proposed c-Myc induced these epigenetic changes via association with various HATs. Moreover, bivalent domains in ESCs coincide with binding of at least one of the embryonic pluripotent transcription factors (Oct4, Nanog and Sox2), and often with a combination of them [57,58]. One way to induce reprogramming is thus to treat reprogramming cells with HDAC inhibitors (TSA and VPA) in order to reset the histone modifications to accelerate reprogramming [51]; although HDAC inhibitors were also shown to cause apoptosis in MEFs [59,60]. Accordingly, treatment with VPA can serve to replace c-Myc, which was documented to recruit HATs [61,62]. Another small molecule triggering epigenetic modifications, BIX-01294, which inhibits the histone methyl transferase G9a, was similarly found to enhance reprogramming [50]. Finally, an additional mechanism by which vitamin C treatment induces reprogramming could be via enhanced activity of histone demethylases, since vitamin C is a co-factor of those enzymes [32,63].

#### 2.2.3. ATP-dependent Nucleosome Remodeling Factors and Other Chromatin Remodeling Complexes

ATP-dependent nucleosome remodeling is another key regulator of pluripotency and reprogramming. The four main subfamilies characterized so far are the CHD, SWI/SNF (also called BAF), ISWI and INO80 subfamilies [37]. For example, down-regulation of Chd1 (Chromodomain-Helicase-DNA-binding protein results) in a strong decrease in MEF reprogramming efficiency. Mechanistically, Chd1 down-regulation results in an increase in the heterochromatin marks, H3K9me3 and HP1γ foci, without affecting the distribution of H3K4/27me3 marks or demethylase expression levels [64]. Altogether these data suggest that in ESCs, the opposing forces of euchromatin and heterochromatin are kept in check through the action of Chd1 that maintains the heterochromatin at a reduced level in pluripotent cells. Chd1 may fulfill this role by mediating the incorporation of the H3 histone variant, H3.3, into nucleosomes, as this histone variant is generally associated with active genes and is less prone to H3K9 methylation [65]. It is interesting to note that transcription factors such as Oct4, Sox2, Nanog and Smad1 (which functions in BMP signaling) can bind to the Chd1 promoter, yielding a clue about how epigenetic remodelers are controlled by the core transcription factors [66].

Using quantitative mass-spectrometry, Singhal *et al.* systematically identified nuclear extract fractions that could reactivate the endogenous Oct4 locus [67]. MEFs treated with a specific fraction of nuclear extracts activated endogenous Oct4 expression in only eighteen hours, in contrast to the three weeks needed when using retroviral infection by OKMS. They identified the active factor as the ATP-dependent SWI/SNF (also called BAF) chromatin-remodeling complex (Brg1, Baf155). This complex synergizes with OKMS to enhance genomic demethylation and enhances reprogramming efficiency [67]. Addition of Brg1 and Baf155 predominantly increased the active chromatin mark H3K4me3, rather than changing the repressive H3K27me3 mark. This sheds light on the mechanism by which the esBAF complex (BAF complex regulating pluripotency in ESCs) regulates the expression of numerous genes involved in self-renewal, proliferation and differentiation potential [68,69].

The Polycomb group (PcG) is a group of transcriptional repressing protein complexes comprised of two main complexes, PRC1 and PRC2. Pereira *et al.* showed that expression of both PRC1 and PRC2 components in the mouse ESCs was required for reprogramming to occur when heterokaryons were used (in this case mouse ESCs fused with human lymphocytes) [70]. PRC2 depends on the activity of three core proteins that catalyse H3K27 methylation: Eed, Suz12 and Ezh2. Knock-down of each of those components revealed that they were critical for the proper function of PRC2 during reprogramming [70]. Furthermore, the PRC2 complex triggers the recruitment of PRC1, which blocks transcription by multiple mechanisms (reviewed in [71]). Indeed, Pereira *et al.* highlighted that PcG is essential for the repression of differentiation markers in somatic cells to allow proper reprogramming, and has non-overlapping functions with other transcription repressive complexes.

Finally, UTF1, which is a chromatin-binding transcriptional repressor that interacts with histone tails and recruits nucleosome remodelers, was also involved in the regulation of reprogramming [25]. Selecting for human ESCs expressing UTF1 ensured a better quality of the stem cell population upon passaging [72]. Moreover, the over-expression of UTF1, which has HAT activity, resulted in a significant increase in the number of reprogrammed colonies and could replace the need for c-Myc during human adult fibroblast reprogramming [25]. Despite its pro-pluripotent role, UTF1 knockdown delays ESCs differentiation [73]. This could be due to the fact that UTF1 represses specific differentiation genes but concomitantly keeps those chromatin regions active and thus ready to be expressed upon induction of differentiation. Another possibility is that UTF1 functions are context dependent and thus depend on other transcription factors. Consistent with the second hypothesis, UTF1 does not activate transcription non-specifically, but rather enhances transcription in an ATF-2 dependent manner [74].

#### 2.2.4. Telomeres, X Inactivation and Gene Imprinting

Maintenance of telomeres is essential to maintain chromosomal stability and prevents senescence. The telomerase complex, responsible for maintaining the telomeres, is active mostly during embryonic development and in adult stem cells [75,76]. Both telomeric and subtelomeric regions possess the repressive heterochromatin domains, H3K9me3, H4K20me3, HP1γ, and highly methylated DNA. In addition, Marion *et al.* showed that H3K9me3 and H4K20me3 heterochromatic marks are decreased during reprogramming, which coincides with a lengthening of telomeres in iPSCs to lengths that match ESCs telomeres. Consistently, early work in reprogramming showed that Tert levels were increased in iPSCs [2,77]. In female somatic cells, one of the X chromosomes is inactivated, while in female pluripotent cells both X chromosomes are activated. During reprogramming of female cells, the inactivated X chromosome (Xi) is reactivated (Xa) [42,78], but it is not clear yet whether reactivated X directly regulates pluripotency or pinpoints cells that have acquired a pluripotent epigenetic state. Finally, genomic imprinting is an epigenetic marking process that causes genes to be expressed depending on their parental origin; with one of the parental chromosomes being silenced via DNA methylation and/or core histone proteins modifications. Generally, imprinted loci are reset during reprogramming [79]. Intriguingly, aberrant methylation of the imprinted Dlk1-Dio3 locus was observed in iPSCs compared to background-matched ESCs, resulting in paternalization of the maternally inherited locus. This was further correlated with a poor ability of iPSCs with silenced Dlk1-Dio3 locus to contribute to the germline after aggregation. Interestingly, this was rescued either by forced expression of *Gtl2*, a gene belonging to this locus, or genomic reactivation of this locus after treatment with the histone deacetylase inhibitor VPA [79]. This suggests that the Dlk1-Dio3 locus regulates the developmental potential of iPSCs. It is worthy to note that the Dlk1-Dio3 locus encodes five protein-coding genes, three non-coding RNA, one snRNA and 47 miRNAs. [80]. The regulation of this locus during reprogramming outlines how OKMS may be involved in gene imprinting in germ cells, but the mechanism is yet to be determined.

### 2.3. The Pluripotent Transcriptional Circuitry

The large changes in the epigenetic landscape of reprogramming cells points to major reorganization of gene expression as cells move between differentiated and pluripotent states and re-establishment of a stable self-sustaining transcriptional network that maintains pluripotency is key for successful reprogramming. Extensive efforts have been applied to decipher how transcription factors regulate the pluripotency expression program. This has revealed that Oct4, Sox2 and Nanog are the major core transcription factors that maintain pluripotency of both mouse ESCs and cells in the ICM of the blastocyst. Thus, deletion of the *Oct4* or *Nanog* genes leads to a loss of pluripotency with ectopic differentiation towards trophectoderm or extraembryonic endoderm, respectively [81–83]. *In vitro*, knock-down of either Oct4, Sox2 or Nanog, results in immediate changes in gene expression in mouse ESCs when compared to the knock-down of other pluripotency-associated transcription factors, such as *Esrrb* [84]. Moreover, Oct4, Sox2 and Nanog bind to and regulate each other's promoters, which results in an enforced feedforward-feedbackward system that sustains robust expression of these genes [57,58]. Furthermore, many of the genes associated with ESC pluripotency (*i.e.*, *Klf2*, *Klf4*, *Esrrb*, *Sall4*, *Tcl1*, *Tbx3*, *Dppa4*) are regulated by at least two out of three of Oct4, Sox2 and Nanog [57,58,84,85]. Interestingly, genome-wide analysis of genomic elements bound by these core transcription factors compared with the gene expression profiles in mouse and human ESCs identified numerous promoters that are occupied but not expressed. Functional analysis of many of these genes revealed that they are involved in lineage commitment (*i.e.*, *Eomes*, *Lhx5*, *Myf5*, *Gata6*). In addition, ChIP analysis of genes bound by the Polycomb Group (PcG) showed marked co-occupancy of such repressed promoters by PcG together with Oct4, Sox2 or Nanog, both in human and mouse [56,86,87] and revealed that Oct4 also binds PcG, as do other pluripotency-associated factors, such as Klf5, Sall4 and Esrrb [69]. This underlines the dual role of the core transcription factors in both maintaining the expression of pluripotency genes, while preventing the expression of differentiation genes [88,89]. More evidence linking the regulation of epigenetic state with the core transcription factors Oct4 and Nanog was revealed by the discovery that Oct4 and Nanog interact with multiple other components of chromatin remodeling complexes, such as NuRD, SWI/SNF, Sin3A-HDAC complexes and the transcription repressor and demethylase LSD1 [90]. Components from those chromatin-remodeling complexes were further found to be associated in complexes together with the core transcription factors, by immunoprecipitation coupled to mass spectrometry [91]. The functional importance of these chromatin remodelers was highlighted through a genome-wide RNAi screen in human ESCs; where multiple components belonging to chromatin remodeling complexes were found to impair the pluripotency of human ESCs [92]. The tight interaction of the chromatin remodeling complexes with the core transcription factors places them within the transcriptional network regulating pluripotency and unveils another level of complexity in the reprogramming process. Pluripotency-related miRNA (*i.e.*, miR-290 and miR-302 clusters) also regulate reprogramming through suppression of differentiation-related miRNA (*i.e.*, let-7 family, miR-21) [93], with Oct4 and Nanog directly regulating their expression [93]. On the other hand, differentiation-related miRNAs (*i.e.*, miR-296, miR-470) can inhibit the expression of the core transcription factors, creating a mutually-exclusive loop of developmental- or pluripotency-related regulators (reviewed in [94]).

**Figure 2:**
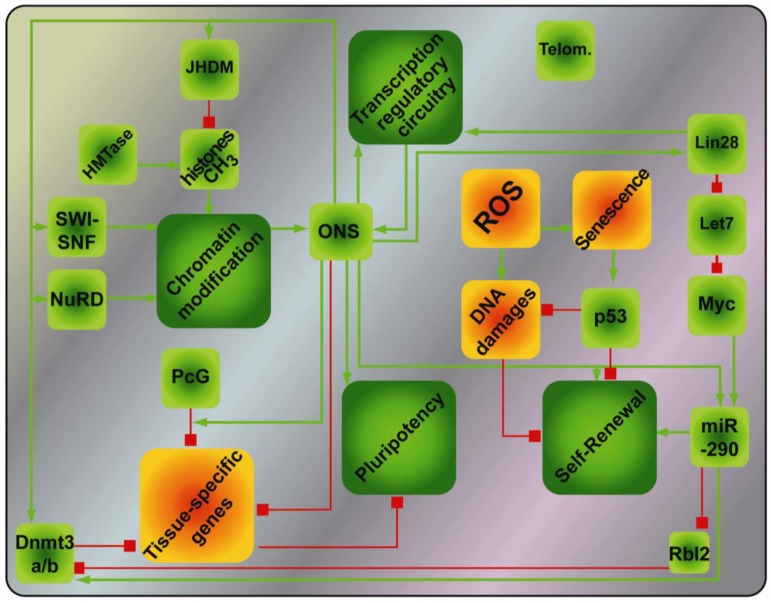
Interactions between the different cellular processes that regulate pluripotency. Green arrow: positive interaction, Red arrow: negative interaction. ONSK = Oct4, Nanog, Sox2; PcG = Polycomb complex; HMTase = Histone methyl transferase; ROS = radical oxygen species.

Extensive efforts have thus uncovered a complex and sophisticated network of events regulating pluripotency that gravitate around the core transcription factors (Figure 2). As a result of these mechanistic studies, novel combinations of reprogramming factors, *i.e.*, OS + Esrrb or OKS + miR-291-3p [20,95], or more efficient combinations, *i.e.*, OKMS + Nanog [17] have been uncovered. From these insights, a persistent common denominator for reprogramming emerges in Oct4, which cannot be substituted except by his upstream transcriptional regulator, the orphan nuclear receptor Nr5A2 [96].

### 2.4. Pluripotency and the Epithelial/Mesenchymal Identity

In recent years, interaction of the gene expression program with molecular pathways controlling cell morphology and polarity has emerged as a key determinant of cell fate choice and the pluripotent state. In particular transitions between epithelial and mesenchymal have emerged as key events at the heart of multiple primordial biological processes, such as the epithelialization of mesodermal cells through mesenchymal-to-epithelial transitions (MET) during somite segmentation or epithelial-to-mesenchymal transitions (EMT) that underlie gastrulation. Aberrant MET *versus* EMT are also critical in cancer, where EMT promotes metastatic phenotypes, is associated with poorer prognosis in patients and promotes acquisition of stem cell-like properties. More recently, analysis of embryonic stem cells and reprogramming systems has revealed key roles for epithelial *versus* mesenchymal identity in the acquisition and maintenance of pluripotency.

Cadherins are involved in adherent junction establishment between cells, junctions that are essential to delimit tissues or regulate tissue permeability. E-Cadherin (Cdh1), the epithelial cadherin, is involved at the very beginning of development, as embryos lacking both zygotic and maternal Cdh1 do not undergo compaction [97]. Knock-down of Cdh1 in mouse ESCs impairs their pluripotency by facilitating their differentiation, but without affecting self-renewal, proliferation or apoptosis [98]. Moreover, *Cdh1* is critical to reprogram mouse EpiSCs or FAB-SCs (stem cells isolated at the same stage as mouse ESCs, but in media containing Fgf2, GSK3β inhibitor and Activin A) into mouse iPSCs [98,99]. Interestingly, Cdh1 expression is also important during specification of Primordial Germ Cells (PGC), probably helping to maintain an islet of PGCs in a sea of mesenchymal cells [100]. This further confirms the importance of gaining and maintaining “epithelial-like” characteristics in other stem cell populations and suggests that the epithelial-like phenotype of stem cells strongly influences their cell fate and thus might be linked to the core regulatory network controlling pluripotency. Klf2 and Klf4 are candidates linking epithelial-like cell junctions and pluripotency, as they were shown to trigger the reprogramming of mouse EpiSCs to mouse iPSCs, and as Klf4 can directly regulate Cdh1 expression [101–104]. However, whether direct physical interactions between epithelial junctional components might regulate the activity of the core pluripotency transcriptional regulators is unknown. In contrast to the association of the epithelial phenotype with pluripotency, mesenchymal markers are associated with differentiated cells. For example, Zeb2, one of the main transcription factors that regulates the mesenchymal expression profile, is expressed when human ESCs or mouse EpiSCs are differentiated towards neuroectoderm [105].

During the reprogramming of MEFs, a time-series microarray analysis has shown that there is a strong and early switch in expression of genes associated with the mesenchymal program towards an epithelial gene expression program [106]. Mesenchymal associated transcription factors *Snail*, *Slug*, *Twist*, *Zeb1* and *Zeb2* were strongly down regulated, while *de novo* expression of the epithelial markers *Cdh1*, *Epcam*, *Crbs3* and *Occludin* was observed. Concomitantly, miR-200 microRNA family members, which are involved in maintenance of epithelial cells and stem cells fate [107,108], were also strongly induced early during reprogramming [106]. Concomitant reorganization of actin stress fibres into cortical actin together with acquisition of epithelial adherens junctions and aspects of apical-basal polarity similar to ESCs confirmed that MET is one of the earliest events in reprogramming. In addition, knock down of *Cdh1* or over-expression of *Snail* prevented reprogramming [104,106], while expression of miR-200 mimics induced MET in MEFs, and accelerated their reprogramming upon re-expression of OKMS [106]. Thus, MET is a key event required during MEF reprogramming. Consistent with these findings, live cell imaging has revealed that reprogramming MEFs change their morphology from flat and elongated cells to packed, small and cuboidal cells that also increase their proliferative rate. All the cells that were later shown to be positive for Cdh1 and Nanog underwent these morphological changes [109]. The importance of MET during human cell reprogramming was further highlighted when published microarray data sets were re-analyzed by Wang *et al.*, who found that false-positive colonies (*i.e.*, cells that aggregate in colonies but without expressing alkaline phosphatase) did not undergo MET [110,111]. As the morphological and epithelial gene expression signature of MET all occur before the onset of expression of the earliest pluripotency-associated markers [104,106], MET is one of the earliest events driving reprogramming.

The core pluripotency factors also may contribute to maintenance of the epithelial phenotype in embryonic stem cells, since they can repress the mesenchymal program, for example by down-regulating *Snail* and *Zeb1* [104,105]. However, Chng *et al.* found opposite results concerning the role of Sox2, showing that Sox2 induced the expression of *Zeb1*, although this was after Oct4 and Nanog were silenced [105]. As Zeb1/Zeb2 are targeted by miR-200 family, which is associated with epithelial identity [107,112], but at the same time repress miR-200 family expression in a double negative feedback loop [113], it will be interesting to study whether the first event of MET is loss of Zeb1/Zeb2 expression or the gain of miR-200 family expression. Reprogramming of MEFs to iPSCs brings a new case where stemness and cell fate is associated with the epithelial-mesenchymal plasticity. The role of mesenchymal and epithelial proteins during acquisition or maintenance of stemness is of particular interest as the studies in embryonic stem cells stand in stark contrast to cancer, where stem cell like properties are driven by EMT [114].

### 2.5. Regulation of the Reprogramming/Pluripotency by Extracellular Signals

Cells make a variety of cell fate decisions in response to a diverse range of extracellular cues. Integration of inputs from cytokines, hormones and interactions with other cells or with the extracellular matrix, will thus trigger signaling cascades that will intersect with the transcriptional network to regulate various aspects of pluripotency. Of particular interest here are the Leukemia Inhibiting Factor (LIF)-Stat3 pathway, Wnt-βcatenin signaling and the Transforming Growth Factor beta (TGFβ)- and related Bone Morphogenetic Protein (BMP)-Smad transcriptional regulators. LIFs signaling via the transcription factor Stat3 and have long been known to promote pluripotency of mouse ESCs, but may have no role in human ESCs and mouse EpiSCs. LIF-Stat3 signaling is also essential for mouse reprogramming [115] and ChIP-Chip analysis has demonstrated that Stat3 is co-localized with Nanog and Oct4 on many promoters. In particular, Oct4 and Stat3 co-occupy and regulate expression of Klf2 and Klf4 [102,116]. Wnt-βcatenin signaling is also important in mouse ESCs pluripotency [117] and genetic or chemical inhibition of GSK3β, which is a key negative regulator of Wnt-βcatenin signaling, strongly promotes pluripotency in mouse ESCs. TGFβ family members on the other hand have complex roles in stem cell biology, both promoting pluripotency and controlling cell fate that is highly dependent on cell type [118]. Thus, in human ESCs and mouse mesenchymal stem cells, TGFβ signaling, acting via Smad2/3 in conjunction with the TAZ transcriptional co-modulator, is required to maintain pluripotency [119,120]. More precisely, in human ESCs and mouse EpiSCs, TGFβ-Smad2/3 promote Nanog expression and associate with the Nanog promoter [121–123], but the Smad-factors supporting this interaction remain unidentified. On another hand, in mouse ESCs, the BMPs, signaling via the Smad1/5/8 pathway, play a key role in pluripotency in a tightly regulated balance with LIF [124]. LIF inhibits pro-neuronal differentiation by inhibiting MAPK signaling, while BMP signaling stimulates expression of the *Id* genes (*Inhibitor of differentiation* [124]). Interestingly, over-expression of Nanog bypasses the requirement for BMP4 signaling or Id expression, suggesting that Ids and Nanog, or Nanog targets, might repress common genes [124]. These studies thus establish sets of extrinsic cues that conspire to regulate pluripotency in a contextual manner; in mouse ESCs, BMP, LIF and Wnt ligands maintain pluripotency, while FGF2 and TGFβ/Activin are employed in human ESCs and mouse EpiSCs. Moreover, by manipulating signaling pathways using small molecule kinase inhibitors, pluripotent states can be made inherently stable to reveal a ground state of pluripotency. In particular GSK3β inhibitors, which can potently upregulate Wnt-βcatenin signaling, together with inhibitors of the MAPK pathway, can promote pluripotency in a number of systems [117].

Wnt signaling in addition to promoting stemness in mouse ESCs can enhance reprogramming [125], possibly by promoting proliferation, since it also increases proliferation of human ESCs [126]. Accordingly, c-Myc was reported as a Wnt target gene, and the effect of activating Wnt signaling during reprogramming are only apparent in the absence of the c-Myc transgene [125]. At the molecular level, one of the downstream targets of Wnt signaling is the transcription factor Tcf3. Tcf3 has an intriguing role in pluripotency, as it can down-regulate *Oct4*, *Nanog*, *Sall4*, *Sox2* [127], but is still required for pluripotency and reprogramming [106,128] and has functional interactions with another transcription factor called Tbx3. Interestingly, while adding Tbx3 to the cocktail of reprogramming transgenes (OKS) during MEF reprogramming improved germ line transmission by the resulting iPSCs [129], the same study showed Tcf3 inhibited Tbx3, apparently placing them as antagonists. Tbx3 is also described as a mesenchymal marker in the context of primordial germ cells specification, and its expression is inhibited by Prdm14, one of the main players of PGC specification in mouse, or pluripotency maintenance in human ESCs [92,100]. Tcf3 and Tbx3 are therefore appearing to have opposing functions with pluripotency, despite being essential for reprogramming. Tcf3 was demonstrated to be important to maintain the differentiation potential of pluripotent cells, knock-down of Tcf3 in mouse ESCs preventing them from differentiation, but without compromising their pluripotency [130]. This suggests that, similarly, Tbx3 might function by poising the iPSCs, reprogrammed with OKS + Tbx3, for PGCs contribution; the mechanisms involved are unknown. The Wnt pathway also directly interacts with the LIF-Stat3 pathway signaling to increase Stat3 levels, while the LIF pathway promotes Stat3 phosphorylation and activation [131,132]. Similarly, hypoxia, through HIF1α, was shown to regulate Wnt signaling and in mouse ESCs, HIF1α was shown to bind to the promoter and enhance the expression of Lef1/Tcf1, one of the main transcription factors downstream of canonical Wnt-βcatenin [133]. Regulating Wnt signaling could be another mechanism by which hypoxia improves reprogramming efficiency [31].

FGF signaling is also critical in stem cell biology. FGF2 participates in the repression of differentiation-related factors in human ESCs and mouse EpiSCs, for example by inhibiting PAX6, a neuroectoderm-inducing factor, even though the precise mechanism remains unknown [121,123]. FGF2 also inhibits Klf2 [123], which triggers the reprogramming of mouse EpiSCs into mouse ESCs [102], and thus might be a mechanism explaining why inhibition of FGF2 signaling also promotes reprogramming of human ESCs into mouse-ESC like human ESCs [6]. Moreover, KLF4 is one of the factors used to convert human ESCs to mouse-ESC-like human ESCs [7]. Finally, FGF2 also has a role in the feeder culture layer, where it stimulates Activin A production to indirectly induce Nanog expression in human ESCs [134,135].

**Figure 3:**
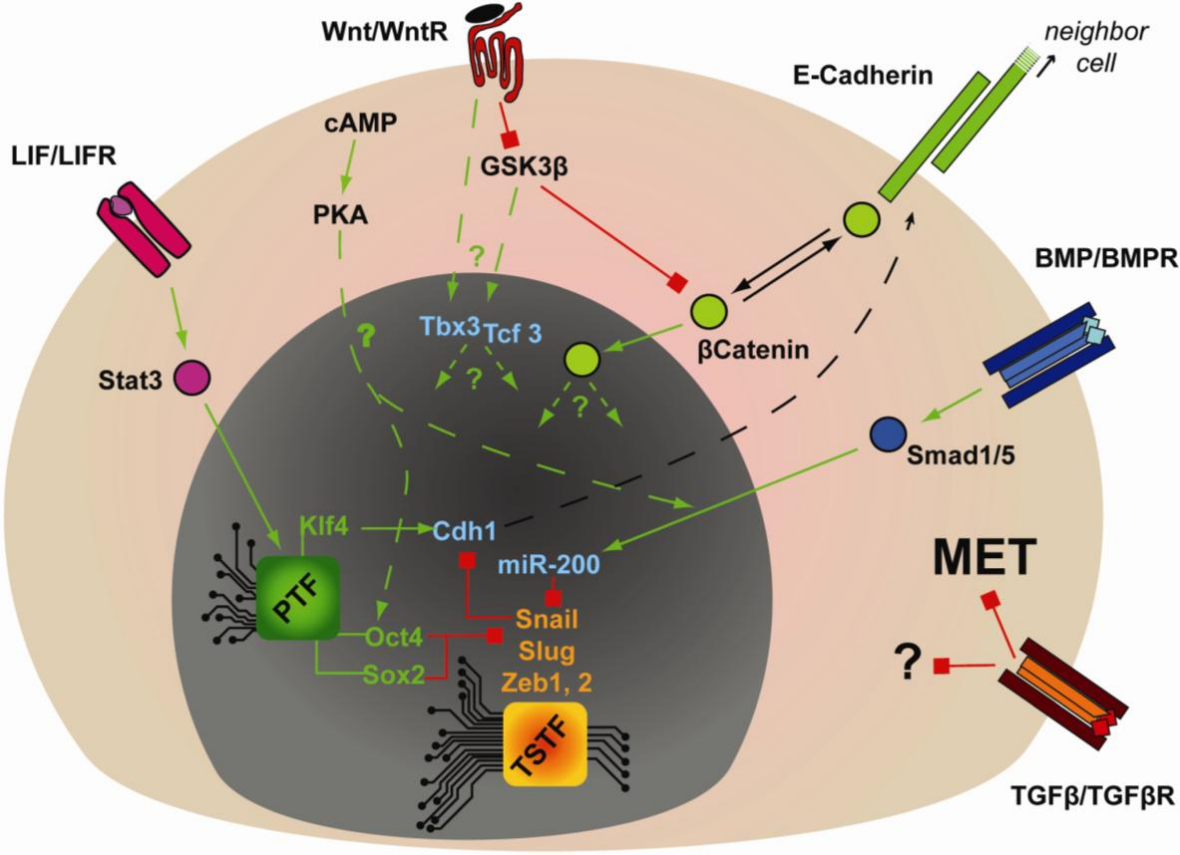
Pathways regulating the acquisition of pluripotency. MET: mesenchymal to epithelial transition, PTF: Pluripotency associated Transcription Factors, TSTF: Tissue Specific Transcription Factors.

Extracellular cues are also critical in regulating the gain and maintenance of epithelial identity that is critical for embryonic stem cell pluripotency (Figure 3). LIF- or BMP-dependent expression of *Cdh1* is critical for reprogramming mouse EpiSCs or FAB-SCs into mouse iPSCs [98,99]. Furthermore, during somatic cell reprogramming by OKMS, BMP signaling was shown to be required for MET and induction of miR-200 family members in a strictly OKMS-dependent manner [106]. According to genome-wide ChIP analysis, Oct4 and Sox2 have the highest percentage of promoter co-occupancy with Smad1 [66]. Moreover, Li *et al.* showed that over-expression of Oct4 and Sox2 inhibited the expression of the mesenchymal transcription factors Snail and Slug [104] and in fibroblasts Klf4 can strongly induce epithelial markers (*i.e.*, *Cdh1*, *Epcam*, *Occludin* and *Cldn3*) [103,104]. These studies implicate a coordinated regulation of MET by multiple components of OKMS, but how OKMS and BMP-Smad signaling synergizes to regulate MET is unknown. Regardless, these pathways may also be relevant in an *in vivo* context, since Cdh1 together with BMP and Wnt signaling are required for the specification of Primordial Germ Cells (PGC) [100].

The role of TGFβ signaling in reprogramming is opposite to that of BMP, since small molecule antagonists of the TGFβ receptor were found to enhance reprogramming of mouse, human and rat cells [110,136–138]. These results might be explained by considering that TGFβ is a potent inducer of the opposite process to MET, namely EMT [139]. However, the antagonistic role of TGFβ might not be simply restricted to preventing MET, since continuous treatment of human fibroblast with TGFβ receptor antagonist, in conjunction with the MEK inhibitor PD0325901, enhanced the retrovirally-induced reprogramming efficiency [110]. This might be related to TGFβ-dependent regulation of cell cycle progression, since TGFβ signaling inhibits the expression of c-Myc, and induces the expression of p21 and p15 [140].

These studies highlight that the context in which cells receive extracellular cues such as LIF, TGFβ and Wnt, has a significant impact on the nature of the biological output. For example, while TGFβ inhibits reprogramming of cells, it is also required to maintain pluripotency of human ESCs and mouse EpiSCs. Therefore, understanding how contextual-dependent responses are conferred in embryonic stem cell systems, to control cell fate, is critical to understanding the mechanisms underlying pluripotency and reprogramming.

### 2.6. Models of Reprogramming

The increasing compendium of knowledge on the reprogramming process is shedding light on the molecular mechanism underlying factor-based reprogramming. Because reprogramming by OKMS can yield various outcomes, such as partially reprogrammed cell populations, and the timing of reprogramming is variable, it has been suggested that reprogramming by OKMS is dependent on stochastic events. One can hypothesize that this may involve stochastic changes in gene expression as described in multiple organisms [141]. However, there is mounting evidence in favor of sequential events occurring during reprogramming, resulting in stepwise progression with multiple transition states. Stadtfeld *et al.* showed that mesenchymal marker Thy1 was lost before ES marker SSEA1 was gained [142]. As noted above, MET similarly occurs prior to the onset of pluripotency markers and analysis of SSEA1/Alkaline phosphatase and *Nanog/Oct4* has revealed a temporal order in their appearance during reprogramming [106,142]. Sequential events were also documented by live imaging of MEF reprogramming by Smith *et al.*, which also revealed a rapid change in growth rate that occurred prior to the onset of Nanog expression [109]. Indeed, genome-wide expression profiling throughout reprogramming [106] revealed changes in expression in thousands of genes and identified three phases into which most genes cluster during MEF reprogramming: (1) initiation phase, characterized by MET; (2) maturation phase, in which the endogenous pluripotency-associated markers Nanog and Oct4 are expressed; (3) a stabilization phase where the remaining pluripotency-associated genes are expressed and which arises after removal of the transgenes. Stochasticism observed in reprogramming systems may therefore reflect the requirement to remodel the entire gene expression landscape in such a coordinated way. Accordingly, Hanna *et al.* showed that if the starting cell population in factor-based reprogramming survives long enough, they all have a chance to reprogram [17]. In contrast, somatic cell nuclear transfer (SCNT) results in 45–50% reprogramming efficiency within four days [143], and fusion of a somatic cell with a pluripotent stem cell results in reprogrammed cells within one day [144,145]. The capacity of intrinsic and extrinsic factors in these disparate systems to synergize with core pluripotency factors to bring about wide-ranging alterations in gene expression may thus underlie the temporal variations observed in reprogramming. For example, demethylases that are abundant in the egg and ESCs may facilitate rapid epigenetic remodeling [46,146]. Once the key cues that synergize with pluripotency factors are identified, one might expect that factor-based reprogramming will be as efficient as the SCNT or cell fusion reprogramming methods.

## 3. Conclusions

Recent studies in a variety of different iPSCs models have started to illuminate the black box of reprogramming. This has led to insight into how changes in proliferation rate, epigenetic rearrangements, and the establishment of a self-sustaining transcription factor network, are established during the reprogramming process. How these intrinsic cues are coupled to extrinsic morphogen signaling pathways further reveals an intimate coupling between cell fate choice and the establishment and maintenance of pluripotency. Identifying the pathways and limiting events that are governing reprogramming will allow us to develop more efficient strategies for factor-based reprogramming, which will better enable the use of human iPSCs for modeling diseases and for the development of cell-based therapeutics. Understanding the molecular mechanisms underpinning reprogramming will not only pave the way for developing transdifferentiation strategies, but will also provide effective means to evaluate the safety and validity of therapeutic iPSCs.
